# Case Report: Management of a pregnancy complicated by a symptomatic macroprolactinoma

**DOI:** 10.3389/fmed.2025.1651834

**Published:** 2025-11-11

**Authors:** Spyridon Topis, Kalliopi Christodoulaki, Maria Riga, Ioannis Arkoulis, Theodoros Karampitsakos, Tatiana Sidiropoulou, Peter Drakakis, Anastasios Potiris, Sofoklis Stavros

**Affiliations:** 1 Third Department of Obstetrics and Gynecology, University General Hospital “ATTIKON”, Medical School, National and Kapodistrian University of Athens, Athens, Greece; 2 Second Department of Anesthesiology, University General Hospital “ATTIKON”, Medical School, National and Kapodistrian University of Athens, Athens, Greece

**Keywords:** prolactinoma, macroprolactinoma, dopamine agonists, cabergoline, diplopia, pituitary adenoma, pregnancy

## Abstract

**Background:**

Prolactinomas are the most common functional pituitary adenomas and may lead to infertility, visual field defects, and neurological impairment. Pregnancy poses unique challenges to women with macroprolactinomas due to the potential for tumor enlargement under the influence of gestational hormonal changes.

**Case:**

We present the case of a 40-year-old woman who conceived through *in vitro* fertilization (IVF) and presented at 38 + 3 weeks of gestation with new-onset diplopia. The magnetic resonance imaging (MRI) revealed a 29-mm hemorrhagic macroprolactinoma causing compression of the optic chiasm. The tumor had previously been asymptomatic and untreated. Management was undertaken by a multidisciplinary team including obstetricians, endocrinologists, neurosurgeons, and anesthesiologists. An elective cesarean section was performed under general anesthesia with careful attention to intracranial pressure control and hemodynamic stability.

**Outcome:**

Delivery was uneventful, with no perioperative neurological or anesthetic complications. The patient was discharged in good general condition and commenced long-term dopamine agonist therapy, with planned close endocrinological and neurosurgical follow-up.

**Conclusion:**

This case highlights the importance of early diagnosis, individualized management, and multidisciplinary coordination in pregnant women with macroprolactinomas. Dopamine agonists remain the cornerstone of therapy and are generally safe during early pregnancy. Meticulous anesthetic planning is essential to optimize maternal and fetal outcomes.

## 1 Introduction

Prolactinomas (PRLs) are the most common type of benign pituitary adenomas associated with hyperprolactinemia, leading to a wide range of clinical manifestations that primarily affect the reproductive and neurological systems. They represent the most frequent hormone-secreting pituitary tumors, accounting for approximately 40% of all pituitary adenomas (1). Microprolactinomas, defined as lesions measuring less than 10 mm in diameter, are often asymptomatic and typically managed conservatively. In contrast, macroprolactinomas, which exceed 10 mm in diameter, are more likely to cause clinically significant complications—particularly during pregnancy, when physiological hormonal changes may promote tumor growth (2). The clinical manifestations of prolactinomas include menstrual irregularities, galactorrhea, infertility, headaches, and visual field defects, especially when the tumor compresses the optic chiasm (3). Diagnosis is based on elevated serum prolactin levels—confirmed by immunoassays such as the Ima2 multimeric assay—and neuroimaging, most commonly magnetic resonance imaging (MRI). The mainstay of treatment remains dopamine agonists (DAs), particularly bromocriptine and cabergoline, which effectively suppress prolactin secretion and reduce tumor size with variable degrees of success (1).

The coexistence of prolactinoma and pregnancy presents a clinical challenge. During gestation, elevated estrogen concentrations induce lactotroph hyperplasia within the pituitary gland, potentially leading to tumor enlargement and compressive symptoms (4). While microprolactinomas rarely cause complications during pregnancy, macroprolactinomas—especially those involving critical structures such as the optic chiasm—require close monitoring and individualized management strategies. Dopamine agonists are recommended as the first-line therapy for women seeking fertility and during early pregnancy. Among them, bromocriptine remains the traditional choice, although cabergoline is increasingly preferred owing to its superior tolerability and efficacy (5). Anesthetic considerations in pregnant patients with intracranial masses are of paramount importance. Pituitary macroadenomas necessitate meticulous anesthetic planning to prevent hemodynamic instability and elevations in intracranial pressure (ICP). General anesthesia is often preferred over neuraxial techniques because of the potential risk of brain herniation or neurological deterioration in the setting of increased ICP (6, 7).

The aim of the present report is to describe the management of a challenging case of macroprolactinoma in a pregnant woman presenting with new-onset diplopia who underwent cesarean section under general anesthesia. This case underscores the importance of early recognition, multidisciplinary collaboration, and individualized therapeutic planning to optimize maternal and fetal outcomes. The report follows CARE guidelines for clinical case reporting.

## 2 Case presentation

A 40-year-old woman, gravida 2 para 1, at 38 + 3 weeks of gestation, presented to the emergency department of “ATTIKON” University General Hospital with new-onset diplopia for further evaluation and delivery planning. Her past medical history was notable for an untreated prolactinoma diagnosed after her first pregnancy, when elevated serum prolactin levels (220 ng/mL) prompted further investigation. At that time, neuroimaging had demonstrated a 15-mm pituitary adenoma. The patient remained asymptomatic thereafter, and no follow-up assessments were performed. The current pregnancy was achieved via assisted reproductive techniques (ARTs) and was complicated by diet-controlled gestational diabetes mellitus. It is noteworthy that neither a complete hormonal profile nor repeat neuroimaging was conducted prior to the initiation of IVF treatment.

**Figure 1:**
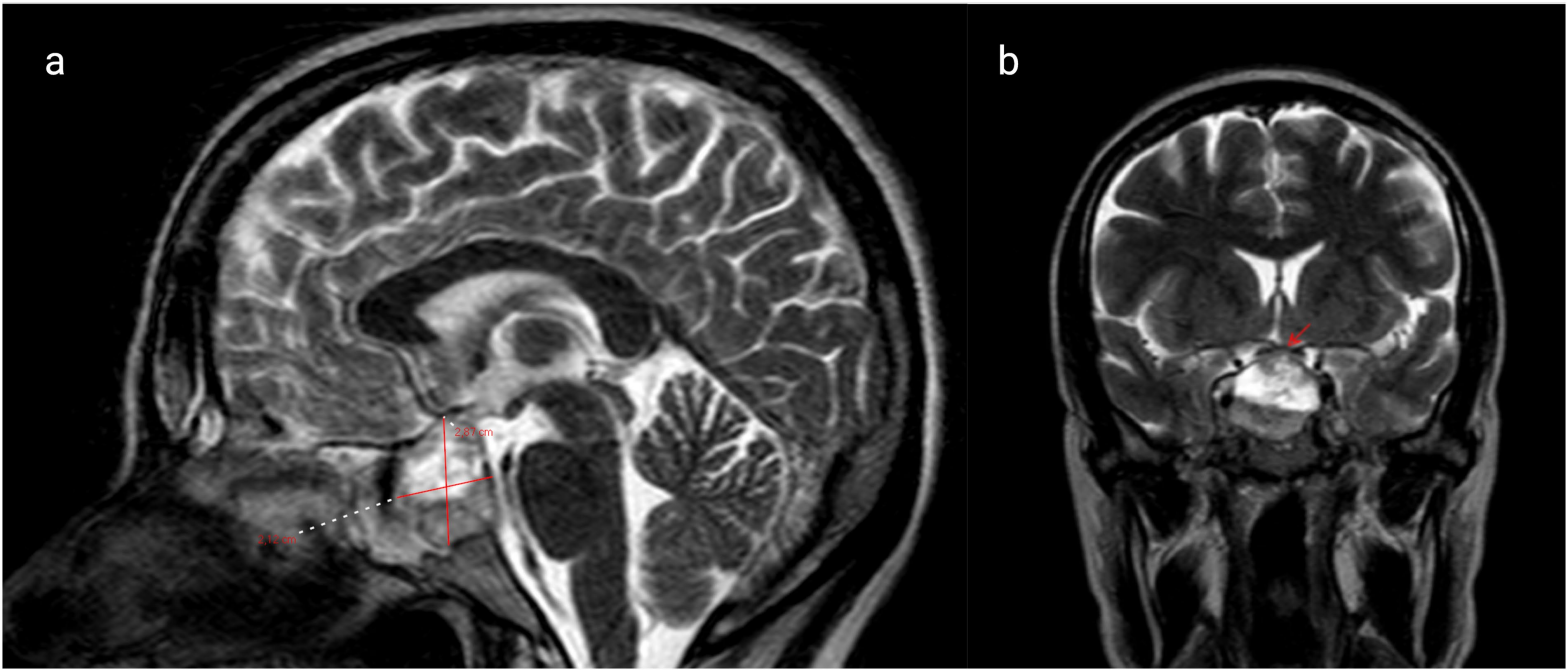
**(a)** Brain magnetic resonance imaging (MRI) reveals a hemorrhagic macroprolactinoma with dimensions of 28.7 × 21.2 mm (marked with red lines) and **(b)** coronal T1 post-contrast MRI shows macroprolactinoma impinging on the right cavernous sinus (marked with red arrow).

Five days before admission, magnetic resonance imaging (MRI) revealed a hemorrhagic macroprolactinoma measuring 28.7 × 21.2 mm (Figure 1a), compressing the optic chiasm and displacing the right internal carotid artery toward the left (Figure 1b). Baseline endocrine evaluation on admission demonstrated normal thyroid function tests (TSH and free T4). Morning cortisol was 12 μg/dL, indicating an intact hypothalamic–pituitary–adrenal axis. Serum prolactin levels were elevated at 310 ng/mL. Ophthalmologic examination confirmed diplopia and mild right-sided ptosis. The pupils were equal and reactive to light. Ocular motility assessment revealed restricted abduction and elevation of the right eye, consistent with partial involvement of the oculomotor (III) and abducens (VI) cranial nerves, while left eye movements were normal. A multidisciplinary evaluation by the neurosurgery and endocrinology teams was undertaken. Neurosurgical consultation recommended expectant management of the tumor, with delivery followed by postpartum reassessment. Initiation of cabergoline therapy was planned after birth.

Given the tumor’s size and suprasellar extension, a comprehensive anesthetic strategy was formulated. A cesarean section was performed under general anesthesia, following established principles for patients with intracranial mass lesions, with an emphasis on maintaining hemodynamic stability and minimizing intracranial pressure fluctuations. Although some literature supports the cautious use of neuraxial techniques in selected cases, general anesthesia was deemed safer in this instance due to the risk of neurological compromise (6, 7). An indwelling arterial line was placed prior to induction for continuous invasive blood pressure monitoring, alongside standard anesthetic surveillance. Lidocaine and remifentanil were administered to attenuate the hypertensive response to laryngoscopy, while esmolol was used for immediate blood pressure control owing to its rapid onset and short duration of action (8). The surgical procedure proceeded uneventfully. The neonate’s Apgar scores were 8 and 9 at 1 min and 5 min, respectively. The patient experienced no intraoperative or postoperative complications. Postpartum diplopia and other visual disturbances resolved completely. The patient was monitored in a standard ward setting and discharged 3 days later in good general condition. She was advised to commence cabergoline therapy, undergo a follow-up MRI at 4 months, and maintain regular endocrinological and neurosurgical surveillance, including serial serum prolactin and macroprolactin measurements.

**Figure 2:**
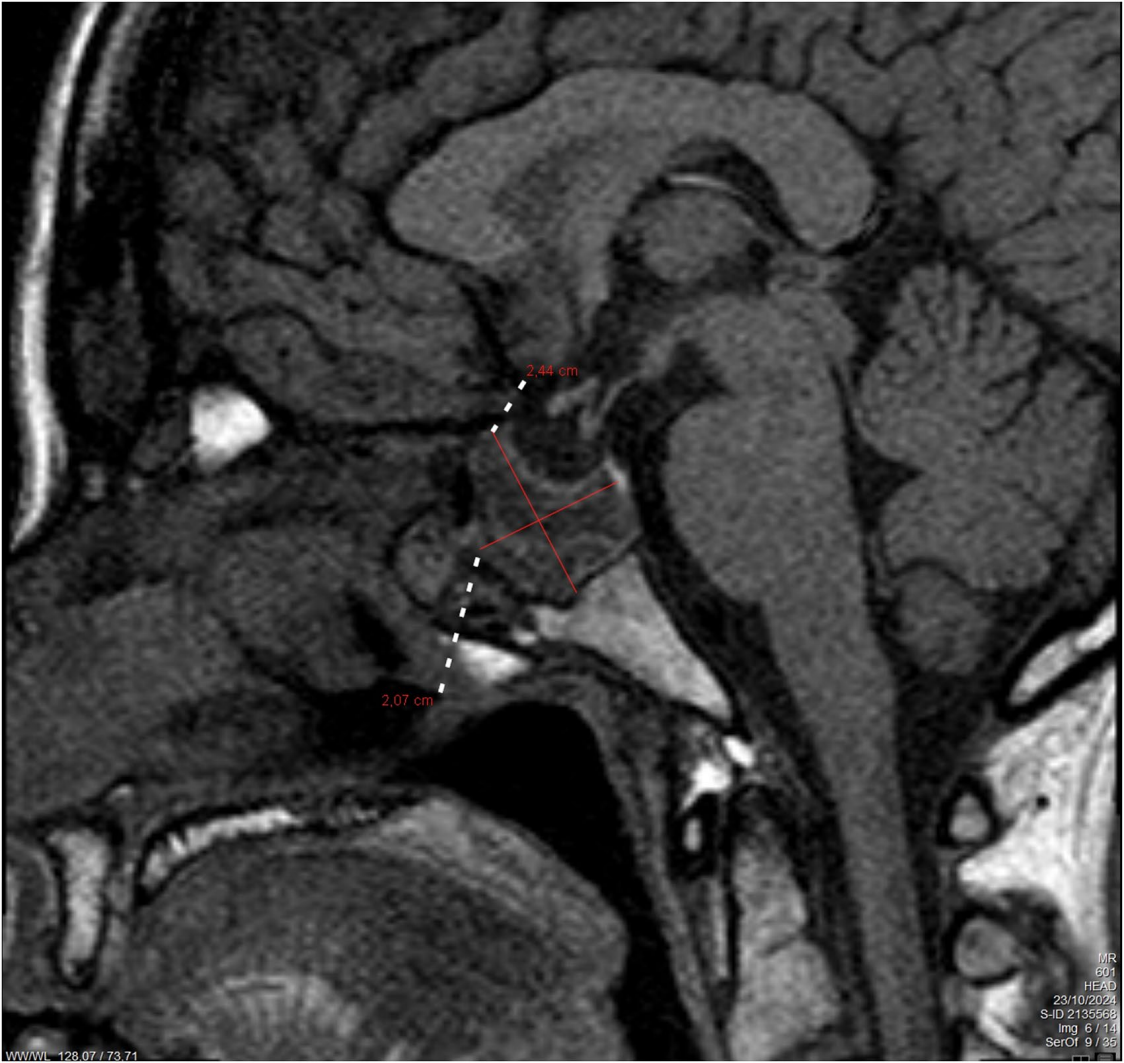
Follow-up brain magnetic resonance imaging (MRI) after 4 months of delivery. The figure shows a slight decrease in the hemorrhagic macroprolactinoma with dimensions of 24.4 × 20.7 mm (marked with red lines).

At the 6-week postpartum review, the patient remained asymptomatic, and serum prolactin levels had decreased to 180 ng/mL. Follow-up MRI at 4 months demonstrated a slight reduction in tumor size, measuring 24.4 × 20.7 mm (Figure 2). Under ongoing multidisciplinary follow-up, she remained stable, with no recurrence of diplopia. A chronological summary of the case is presented in Table 1.

**Table 1:** Timeline of events.

Time point	Event/Intervention	Comments
5 days before admission	Brain MRI examination	Hemorrhagic macroprolactinoma of 28.7 × 21.2 mm
Admission	Onset of diplopia	Neurosurgical consultation, decision to deliver (38 + 3 weeks of gestation)
Admission + 1 day	Cesarean section	Total anesthetic plan with general anesthesia, according to the principles of intracranial mass patients, based on hemodynamic stability and the reduction of elevated intracranial pressure
Admission + 4 days	Patient’s discharge	Stable in good condition
4 months after admission	Follow-up brain MRI	Slight reduction of the macroprolactinoma with dimensions 24.4 × 20.7 mm

## 3 Discussion

The management of macroprolactinomas during pregnancy remains particularly challenging, requiring careful consideration of maternal and fetal wellbeing as well as tumor control. Prolactinomas, including macroprolactinomas, may enlarge during gestation due to estrogen-mediated stimulation. Elevated estrogen concentrations lead to lactotroph hyperplasia, which can promote tumor expansion and precipitate clinical symptoms such as headaches and visual disturbances (4, 9). Symptomatic tumor growth has been reported in approximately 20–30% of pregnant women with macroprolactinomas, particularly when dopamine agonist therapy is discontinued (2). The risk is further increased when the lesion is in proximity to vital structures such as the optic chiasm, as observed in the present case (1).

Dopamine agonists remain the cornerstone of prolactinoma management. Bromocriptine, with its well-established safety profile during pregnancy, is generally regarded as the first-line treatment (1, 3). Cabergoline, a longer-acting agent with better tolerability and efficacy, is increasingly used; however, data regarding its safety in pregnancy remain comparatively limited (5, 10). Current recommendations suggest that, in women with invasive macroprolactinomas or tumors exhibiting suprasellar extension, continuation of dopamine agonist therapy during pregnancy may be necessary to prevent the development of a symptomatic mass effect (9, 11). A recent systematic review encompassing 2,544 pregnancies in women with pituitary adenomas reported miscarriage in 10% of cases, tumor growth in 4%, and congenital anomalies in 2% of those exposed to dopamine agonists (4).

More recent evidence further refines the safety profile of cabergoline in pregnancy. In a 2025 meta-analysis, incorporating 12 studies and 1,387 pregnancies with prolactinoma, fetal loss occurred in 16.1% of cases and congenital malformations in 4.7%. While live birth rates were slightly lower among cabergoline users than among non-users [RR 0.81 (95% CI 0.67–0.98); *p* = 0.03], no significant differences were observed for congenital malformations, preterm birth, or low birth weight (RRs close to 1.0) (12). Similarly, another 2025 systematic review analyzing 1,662 pregnancies exposed to cabergoline during the first trimester found no increased risk of congenital malformations or spontaneous abortion compared with other treatments or untreated controls (13). Collectively, these findings reinforce the relative safety of dopamine agonist use during gestation when the anticipated benefits outweigh potential risks (14).

Transsphenoidal surgery is reserved for cases where medical therapy is ineffective, contraindicated, or poorly tolerated. In pregnant patients with significant visual compromise due to optic chiasm compression, surgical decompression is generally considered safe during the second trimester to minimize fetal risk (1, 2). Furthermore, tumor debulking prior to conception has been associated with a lower incidence of symptomatic enlargement during pregnancy (15).

Anesthetic management in pregnant women with intracranial mass lesions presents additional complexity. General anesthesia is typically preferred over neuraxial techniques due to concerns about elevated intracranial pressure and the risk of neurological deterioration (6, 7). The use of short-acting agents such as remifentanil and esmolol can effectively attenuate hemodynamic fluctuations and the stress response during induction and emergence from anesthesia (8). Nonetheless, isolated reports suggest that regional anesthesia may be safely used in selected patients without evidence of increased intracranial pressure, provided that meticulous monitoring and multidisciplinary coordination are ensured (16). Ultimately, the choice of anesthetic technique should be individualized based on tumor characteristics, neurological status, and anesthesiology team expertise (17).

Postpartum management involves the reinitiation of dopamine agonist therapy, particularly for women who do not intend to breastfeed. Long-term follow-up with periodic MRI imaging is essential to monitor changes in tumor size and guide ongoing care. Serial measurement of serum prolactin and macroprolactin levels facilitates the evaluation of therapeutic efficacy and the early detection of recurrence or progression (1, 3).

## 4 Conclusion

This case underscores the complex management challenges posed by macroprolactinomas during pregnancy and highlights the necessity of a personalized, multidisciplinary approach involving endocrinologists, obstetricians, neurosurgeons, and anesthesiologists. Early recognition, timely initiation of medical therapy, meticulous anesthetic planning, and structured postpartum follow-up are essential to ensure optimal maternal and fetal outcomes. Moreover, this report emphasizes that comprehensive neuroendocrine assessment and appropriate management prior to assisted reproduction could potentially prevent or mitigate such complications. Therefore, heightened clinical vigilance and preconception evaluation should be integral components of care for women with known pituitary adenomas seeking fertility treatment.

## Data Availability

The original contributions presented in the study are included in the article/supplementary material, further inquiries can be directed to the corresponding author.
